# Utilization of Carbon Dioxide and Fluidized Bed Fly Ash in Post-Industrial Land Remediation

**DOI:** 10.3390/ma16134572

**Published:** 2023-06-25

**Authors:** Natalia Howaniec, Janusz Zdeb, Krzysztof Gogola, Adam Smoliński

**Affiliations:** 1 Department of Energy Saving and Air Protection, Central Mining Institute, Pl. Gwarków 1, 40-166 Katowice, Poland; n.howaniec@gig.eu; 2 Department of Research, Technologies and Development, TAURON Wytwarzanie S.A., ul. Promienna 51, 43-603 Jaworzno, Poland; janusz.zdeb@tauron-wytwarzanie.pl; 3 Department of Environmental Monitoring, Central Mining Institute, Pl. Gwarków 1, 40-166 Katowice, Poland; k.gogola@gig.eu; 4 Central Mining Institute, Pl. Gwarków 1, 40-166 Katowice, Poland; 5 Spolka Restrukturyzacji Kopaln S.A., Strzelcow Bytomskich 207, 41-914 Bytom, Poland

**Keywords:** carbon dioxide mineralization, carbonation, fluidized bed fly ash, carbon dioxide utilization, CCU, waste valorization

## Abstract

The utilization of carbon dioxide and combustion products in cost- and energy-efficient technologies is an important element of creating sustainable energy systems, particularly in the transition period towards carbon neutrality and in light of the latest political developments, when solid fuels are still competing for a dominant role in securing energy supplies. Within the study presented, bituminous coal-derived fluidized bed fly ash samples of high calcium content, treated using a dry carbonation method under ambient conditions, were tested in terms of their specific properties to determine their usability in the preparation of injection mixtures for the filling of voids after shallow mining activities and other selected geo-engineering techniques. The study goes beyond the existing literature in terms of the carbonation method used, alkaline earth metal source, scale of the experiment, process conditions employed and product application studied. The results showed that the bituminous coal-derived fluidized bed fly ash, carbonated using the direct method adopted, may be successfully employed as the main solid component (over 82% *w*/*w*) of the injection mixtures for filling voids after shallow mining activities. The achievable compressive strength of a few MPa makes these materials applicable also in terms of ground strengthening in case it is required in light of the expected land development options to be employed. All principal materials used in the injection mixtures developed (carbonated fluidized bed fly ash, carbon dioxide, bottom ash) are industrial waste, and the carbonation method employed is simple and performed under ambient conditions, which reduces the required energy and cost input of filling mixture production, avoids the surface waste storage requirements, and contributes to the development of low energy-intensive carbon dioxide utilization and solid waste valorization methods.

## 1. Introduction

The most urgent needs of the energy sector regarding developments in the circular economy include, apart from the development of highly efficient and novel energy generation methods, the optimal mitigation of carbon dioxide emission and volume of combustion products, alongside their effective economic utilization [1,2]. Despite the deceleration of the world’s economy, carbon dioxide concentration in the atmosphere in 2020 reached values that have never been observed before (412.5 ppm) [3]. According to the forecasts of 2021, carbon dioxide emission from the coal-based energy sector was predicted to be raised by 640 MtCO_2_, with a recovered coal-fueled energy production of 480 TWh [3]. In a world striving to achieve carbon neutrality, the significance of coal is still considerable, with an over 30% share in electricity production. Additionally, coal is responsible for the production of over 70% of the related carbon dioxide emissions. The volume of coal combustion products (CCP) according to the latest reports amounted to 1.22 Gt, with the greatest contributions coming from China (46%), India (16%) and Europe (11%, EU-15: 3%) [4]. The average world level of CCP reuse was 63% and for the European countries (EU-15) it was 94%, while the dominant application fields included the manufacturing of concrete and cement (class F fly ash), as well as gypsum, road works and land reclamation. In Poland, the power industry generated fly ash and slags in an amount of 11.5 Mt in 2020 and for carbon dioxide this amount was 117 Mt [5]. 

Coal is likely to defend its position in the energy mix, with 140 GW of new coal-fueled power capacity already being constructed worldwide and another 430 GW expected [6]. Additionally, it has a renewed role in helping to secure Europe’s energy supplies with the serious political developments of 2022, undermining the status quo of oil and gas supplies in most European countries [7]. All of this makes the technology of carbon capture, utilization and storage (CCUS) an important element of circular and zero-emission economies, especially with the wide implementation of carbon capture and storage (CCS) being hindered due to environmental impacts and low social acceptance [8,9].

Therefore, efforts are being made to combine the utilization of these two streams of energy sector waste in one process based on the principle of natural weathering of silicate rocks (reactions 1–3) and of the capture potential of 1 MgCO_2_ per 2–4 Mg of rock [10,11]:

Olivine: Mg_2_SiO_4_ + 2CO_2_ → 2MgCO_3_ + SiO_2_ ΔH = + 89 kJ/mol (1)

Serpentine: Mg_3_Si_2_O_5_(OH)_4_ + 3CO_2_ → 3MgCO_3_ + 2SiO_2_ + 2H_2_O ΔH = + 64 kJ/mol (2)

Wollastonite: CaSiO_3_ + CO_2_ → CaCO_3_ + SiO_2_ ΔH = + 90 kJ/mol (3)

Before its wide implementation, methods to intensify carbon dioxide mineralization process kinetics and making it less energy- and cost-intensive are necessary, especially through reducing the need for the mechanical and chemical pretreatment of alkaline earth metal sources [12]. The latter ones may include slags from the steel industry, solid residues from flue gas treatment, combustion products, as well as cement and concrete wastes [11,13,14,15]. These materials are, however, even more complex both chemically and in terms of mineralogical content than the natural rocks, and although their utilization is desirable, the process requires more research on the reaction mechanisms occurring at the solid–gas–liquid interface to increase the kinetics and facilitate the mass transport [16].

The use of combustion products in CO_2_ mineralization concerns mainly ash from conventional boilers fed with lignite [17,18], petroleum coke blended with coal [19,20], a number of wastes [21,22,23] and coal of an undetermined rank [24,25,26,27,28,29].

The chemical and mineralogical properties of fly ash from fluidized bed boilers differ significantly from those of conventional boiler fly ash. The latter ones are described as having a diameter <20 µm, density between 0.54 and 0.86 g/cm^3^, surface area of approximately 0.3–0.5 m^2^/g and a wide range of pH values, from acidic (pH 1.2) to alkaline (pH 12.5) [29]. The shape of the conventional boilers’ fly ash is spherical. Its particles form cenospheres packed with plerospheres and amorphous elements and small amounts of unburnt carbon [29,30]. The structure of fluidized bed fly ash, on the other hand, is irregular and porous. Its structure and properties differ from conventional boilers’ fly ash mainly because of the differences in the combustion process parameters: fluidized bed boilers operate at a temperature range of up to 900 °C, while the conventional boilers operate at 1400–1600 °C. Therefore, in a fluidized bed, no melting occurs but the desulphurization process residues are present, contributing to, e.g., a relatively elevated content of free calcium oxide [29]. This fact creates new challenges in terms of the recognition and application of the techniques stabilizing fluidized bed fly ash properties, treatment with these techniques and their utilization in conventional and novel applications. An increased content of free calcium oxide (over 1% *w*/*w*) may be disadvantageous in terms of fluidized bed fly ash utilization in geo-engineering, underground mining techniques and road works. It also introduces the risk of their classification as hazardous substances, with the relevant codes H315 (causes skin irritation), H318 (causes serious eye damage) and H335 (may cause respiratory irritation) [31,32]. The process of carbon dioxide mineralization with the use of fluidized bed fly ash converts free calcium oxide into a stable and more environmentally friendly carbonated form, reducing the negative impact of untreated fluidized bed fly ash on human health and the environment. Furthermore, it makes use of carbon dioxide, reducing its emission. In this way, the process combines utilization and valorization of two considerable energy-sector-derived waste streams [31,33].

The main routes of carbon dioxide mineralization with the use of a waste-derived alkaline earth metal source include direct wet carbonation, with carbon dioxide dissolved in water solution; indirect wet carbonation, with the chemical extraction (with the use of acids or ammonia salts solutions) of alkaline earth metal ions and carbonation of the resulting leachates; and direct dry carbonation in the gas–solid system. Most of the works on fly ash carbonation have been reported to use the complex and energy-demanding, two-stage indirect wet method [17,18,24,25,26,29,34] and some use the dry method with increased pressure and temperature, as well as optional steam dosing [21,22,23,27,30,35,36]. Reports on the dry carbonation method employed at ambient conditions are limited in their number, the scale of experiments and the parameters tested [27], with some recent exceptions in these terms [31,33].

Furthermore, although the use of untreated fly ash from conventional boilers in the handling of voids resulting from the mining of minerals and rocks is recognized in the literature, it is focused mainly on the flow properties of filling mixtures in pipeline transport and site-specific or modelled filling effects [37,38,39,40]. Some works involve more extensive studies of the chemical, hydraulic and mechanical properties of backfilling mixtures prepared with the addition of only a limited amount of conventional fly ash [40,41,42,43,44,45], or the use of unconventional materials or treated waste materials, like temperature-treated coal refuse as a component of blends with conventional fly ash in backfilling mixtures [43], limestone aggregate with conventional fly ash and low pozzolanic cement [46], or waste glass granulated blast furnace slag and fumed silica [47]. Most of the research is devoted to the cemented paste backfill composed usually of coal gangue, binders (e.g., Portland cement), slag and conventional fly ash [42]. In such mixtures, fly ash is used typically in the amount of 20–30% *w*/*w* [37,42] with a Portland cement addition of up to 10% *w/w* to reach a maximum compressive strength of approximately 5 MPa [42].

The extensive studies on the physical, chemical and rheological properties of injection mixtures for filling voids after shallow mining activities, and based on fluidized bed fly ash application as the dominant component (approx. 90% *w*/*w*), are missing from the literature. Similarly, the experimental data on the effects of dry carbonation process on the properties and the resulting applicability of carbonated fluidized bed fly ash in the preparation of mixtures for mining engineering and land reclamation are not reported in the literature. 

Within the study presented in this paper, bituminous coal-derived fluidized bed fly ash samples of a high calcium content treated using an innovative approach of a dry carbonation method were tested in terms of their specific properties and usability in the preparation of filling mixtures for the filling of voids after shallow mining activities and other mining geoengineering techniques. Fluidized bed fly ash samples differing in regard to their physical and chemical properties, and with increased free calcium oxide content, were treated using a carbonation process at a large laboratory scale. Next, samples of filling paste were prepared and tested in a wide range of parameters covering density, flowability, the amount of the overlaying water, setting time, slakeability, viscosity and compressive strength after 28, 56 and 84 days. The novelty of the study concerns, therefore, the carbonation approach adopted in terms of the process conditions and scale of its implementation; the use of bituminous coal-derived fluidized bed fly ash boilers in the CO_2_ mineralization process; fluidized bed fly ash valorization through improving the environmental safety and property stabilization; as well as testing the resulting material as the main solid state component (over 82% *w*/*w*) of injection mixtures, within a wide range of parameters, determining its applicability in various geoengineering techniques. As such, this study contributes to the development of less energy-intensive carbon dioxide utilization and solid waste valorization methods.

## 2. Materials and Methods

### 2.1. Materials

Fluidized bed fly ash samples supplied by TAURON Wytwarzanie S.A., the leading power provider in Poland (denoted 1–4 in Table 1), carbon dioxide gas grade of 99.99% and tap water were used in the direct dry carbonation method adopted for the conversion of fluidized bed fly ash to a stable and more environmentally friendly carbonated form [33] (denoted 1C–4C, respectively), being the main solid-state component of the filling mixtures tested. The bottom ash, provided also by TAURON Wytwarzanie S.A. and sieved to a grain size below 2 mm, cement of the strength class 42.5, as well as a commercially available mineral mining binder (denoted 5–7, respectively) were applied as solid additives in the preparation of the selected filling mixtures tested. The physical and chemical characteristics of the materials are given in Table 1.

The main metal oxide components of the fluidized bed fly ash tested were silicon, aluminum and calcium (Table 1), amounting in total to approximately 71–90% *w*/*w*. The dominant oxide compound of the untreated and carbonated fluidized bed fly ash and the bottom ash was the silicon oxide (approx. 33–51% *w*/*w*), and, in the case of the remaining materials, the calcium oxide (approx. 40–64% *w*/*w*). 

### 2.2. Methods

#### 2.2.1. Carbonation 

The carbonation process was performed based on the patent description [48] and process optimization works were performed within the R&D project [49] in a rotating reactor operated in a batch mode at room temperature and pressure. The volume of the reactor was 0.15 m^3^ and the mass of fluidized bed fly ash applied was 25 kg per batch. Water in the amount of 5% *w*/*w* of ash mass was provided at the start of the process and carbon dioxide was supplied continuously to the reactor with a flow rate of 18 dm^3^/min. The duration of the process was 90–120 min depending on the initial free calcium oxide content in fluidized bed fly ash tested to be reduced to the required level, below 1% *w*/*w*. 

#### 2.2.2. Filling Mixtures Preparation and Characterization

In the research study on the application of carbonated fluidized bed fly ash as the main solid state component of injection mixtures for filling voids after shallow mining and other geoengineering techniques, the water mixtures of the composites presented in Table 2 were prepared.

**Figure 1:**
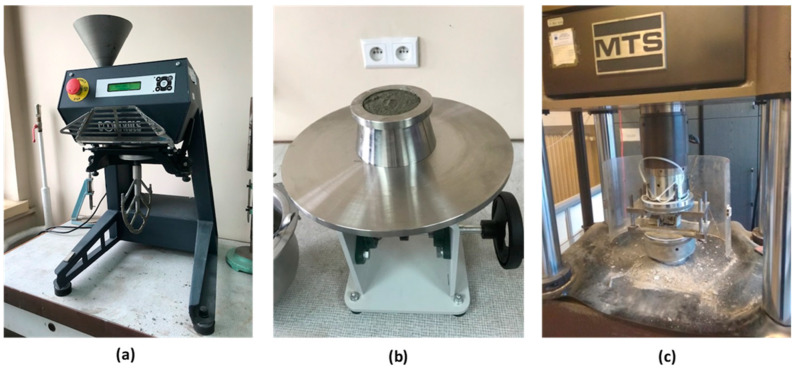
The main elements of the experimental stand for injection mixture preparation and testing: (**a**) mixer B1510FK (AGrIMotor Ktt, Budapest, Hungary), (**b**) device for flowability testing MMC-002 (Multiserw-Morek, Brzeźnica, Poland) and (**c**) material testing system MTS-810 for compression strength measurements (MTS, Eden Prairie, MN, USA).

As the starting point of the research, the composites with a 95% *w*/*w* content of carbonated fluidized bed fly ash and 5% *w*/*w* of cement were prepared. The amount of water added to the composite to prepare injection mixtures, denoted M1–M4, was adjusted experimentally to achieve a minimum flowability of 160 mm. The filling mixtures were prepared with the use of a mixer B1510FK (AGrIMotor Ktt, Budapest, Hungary) and tested in terms of the following parameters: density, flowability, the amount of the overlaying water, setting time, slakeability, viscosity and compressive strength. The main elements of the test stand are presented in Figure 1.

The density of the filling mixtures was determined according to the standard [50] by measuring the weight of 1 dm^3^ of a mixture sample. The flowability was assessed by filling the measuring cone with the mixture tested, followed by its quick emptying, resulting in the spillage of the mixture on a flat, glass surface with the value of the flowability being the diameter of the mixture spilled over the surface. The amount of the overlaying water was measured as the volume of the supernatant in a measuring cylinder filled with the mixture tested, according to the standard [50]. The slakeability was determined by the assessment of the state of mixture samples of the dimensions 4 × 4 × 16 cm, seasoned for 28 days in a climatic chamber and next soaked in water for 1 day, according to the standard [50]. The measure of the slakeability was expressed as “-” meaning the sample was liquefied or “+” meaning the sample was not liquefied. The setting time was measured with the application of the modified Vicat device by the determination of the depth of immersion of the penetrator of the surface area of 1 cm^2^, under a pressure of 5 kg of the moving part of the device. The setting time is the time needed for the immersion of the penetrator to a depth lower than 3 mm [50]. The viscosity was measured with the use of a ford cup of standardized dimensions placed in a tripod holder and leveled. The ford cup with a closed orifice was filled with the mixture tested, and next the orifice was opened and the time needed for mixture outflow was measured. The value of the viscosity was determined by comparing the time needed for mixture outflow with the time needed for the outflow of water. The cylindrical samples for compressive strength measurement were prepared and seasoned in the climatic chamber according to the standard [50]. On the basis of the results reported for filling mixtures M1–M5, further studies were performed with carbonated fluidized bed fly ash sample 4 as the main solid state components and with the application of 2.5% *w*/*w* and 7.5% *w*/*w* of cement (denoted M5–M8); 2.5% *w*/*w*, or 5% *w*/*w* or 7.5% *w*/*w* of mineral mining binder (M9–M14); as well as 2.5–7.5% *w*/*w* of mineral mining binder and 10% *w*/*w* of bottom ash (M15–M20) to assess the effects of particular additives on the carbonated fluidized bed fly ash-based injection mixture properties.

## 3. Results and Discussion

The results of the analysis of water extracts of the materials tested showed that the concentrations of all the heavy metals tested were below the detection limits (Table 3) [51]. This may be related to the alkaline pH of water extracts, which is beneficial in terms of heavy metal immobilization and helps with avoiding the acid mine drainage effect consisting of the formation of sulfuric acid and iron hydroxide in the reaction of pyrite with oxygen and water [37]. The sulfate contents were typical for fluidized bed ash, and acceptable in the practice of coal combustion product utilization in mining techniques.

All materials applied in preparation of filling mixtures also met the requirements of the total radioactive nuclide concentrations: ^226^Ra + ^228^Ra and/or ^224^Ra below 10^4^ Bq/kg (Table 4) [50].

The results reported for the injection mixtures tested in terms of the parameters determining their density, flowability, the amount of the overlaying water, setting time, slakeability, viscosity and compressive strength are given in Table 5.

The results were assessed in light of the parameters determined in the standard [50]; the practical selection criteria for injection mixtures for filling voids and goafs, and for injections in porous and loose layers (Table 6); and in light of the requirements that need to be met by the fine-grained mixtures applied in underground mining techniques (Table 7) [52].

The values of the flowability and overlaying water for all injection mixtures tested met the requirements of the standard [50] and amounted to ≥1.2 Mg/m^3^, ≥90 mm and <15%, respectively (Table 5). None of the samples tested were affected by soaking in water for 1 day after 28 days of seasoning in a climatic chamber. This means that all mixtures prepared based on the carbonated fluidized bed fly ash tested met the requirements of the standard [50], regarding the materials used for the grouting of goafs. 

Furthermore, all injection mixtures tested complied with the parameters of injection mixtures for filling voids and goafs as well as porous and loose layers in terms of the flowability and setting time (Table 5 and Table 6). Only two of twenty injection mixtures prepared did not reach the recommended value of the compressive strength after 28 days of seasoning (0.5 MPa), but it should be noted that the respective values increased after 56 days of seasoning and reached values in the range of 0.80–1.06 MPa (Table 5). Injection mixtures of a high compressive strength, of a few MPa, were also proven to be achievable with the use of carbonated fluidized bed fly ash, which widens its applicability range also for the remediation of lands after shallow mining activities in cases when an additional strengthening of the ground is needed to comply with the requirements of the future land development plans. It should be noted that in most cases of fly ash employment in backfilling, with a relatively low amount of fly ash of 20–30% *w*/*w* [37,42] and with a Portland cement addition of up to 10% *w*/*w*, the maximum achievable compressive strength is approx. 5 MPa [42]. This implies that carbonated fluidized bed fly ash may be efficiently utilized in the production of filling mixtures for various geo-technical applications, in high amounts of 82.5–97.5% *w*/*w* of solid components of the product material, and only small amounts of a binder (2.5–7.5% *w*/*w* of cement), with no detriment to the mechanical properties of the product, in particular the compression strength. Most of the injection mixtures tested also complied with the recommended value of the overlying water below 7% (Table 5 and Table 6).

In terms of the parameters recommended to be taken into account when selecting fine-grained mixtures for application in underground mining techniques, all tested filling pastes complied with the recommended compressive strength values for mixtures used for the grouting of old goafs after the roof falling down, for isolating fire areas, as well as for pit shaft and fore-shaft liquidation (0.1 MPa), and most of the injection mixtures tested were also used for the preparation of plugs and packwalls (0.5 MPa) (Table 5 and Table 7). One injection mixture tested complied with the recommended value of flowability of 210–250 mm, applicable specifically for the grouting of old goafs after the roof falling down, and the remaining mixtures were tested for use in other applications of fine-grained mixtures in underground mining techniques (Table 5 and Table 7). All injection mixtures tested also met the recommended value for overlaying water applicable to mixtures used in the grouting of old goafs after the roof falling down and for isolating fire areas (<14%), and most of the mixtures tested complied also with the values recommended for mixtures for the preparation of plugs and packwalls, as well as for pit shaft and fore-shaft liquidation (<7%) (Table 5 and Table 7). 

On the basis of the experimental results of the study performed, it may be concluded that the bituminous coal-derived carbonated fluidized bed fly ash can be successfully applied as the dominant component (97.5% *w*/*w* of solid components, 2.5% *w*/*w* cement) of injection mixtures for filling old goafs after shallow mining activities, with good rheological and mechanical properties (overlaying water <7 %, flowability >160 mm, compressive strength after 28 d >1.0 MPa). Depending on the location of the injection site and the expected land development option in this area, and in a case when it is desirable to increase the compressive strength of the injection mixtures for filling old goafs after shallow mining activities to a few MPa, this may be achieved with the addition of cement in relatively small amounts of 5–7.5% *w*/*w*. The properties of the injection mixtures tested and prepared with the use of the carbonated fluidized bed fly ash as the main solid component were proven as well, making them applicable in both gravity and pressurized filling methods.

## 4. Conclusions

The results of the experimental study performed indicate the following:

- The bituminous coal-derived fluidized bed fly ash carbonated using the direct, dry method adopted may be successfully employed as the main solid component of the injection mixtures for filling voids after shallow mining activities, complying with the requirements of the relevant standard.
- A compressive strength of a few MPa is achievable for injection mixtures produced with the use of carbonated fluidized bed fly ash as the main solid-state component, which makes them applicable also in terms of area ground strengthening after shallow mining activities, in case it is required in light of particular expected land development.
- The injection mixtures produced with the use of bituminous coal-derived carbonated fluidized bed fly ash as the main solid-state component proved to be applicable for filling redundant voids and goafs, as well as porous and loose layers, by complying with the relevant recommended and additional selection criteria in terms of flowability, setting time, compressive strength and overlying water.
- The applicability of the fluidized bed fly ash treated in the carbonation process as the main solid-state component of the injection mixtures is wide and covers, among other things, the following options for fine-grained mixture use in underground mining techniques: grouting of old goafs after the roof falling down, preparation of plugs and packwalls, isolation of fire areas, and pit shaft and fore-shaft liquidation.
- All principal materials used in the injection mixtures developed (carbonated fluidized bed fly ash, carbon dioxide, bottom ash) are industrial waste and the carbonation method employed is simple and performed under ambient conditions, which reduces the required energy and cost input of filling mixture production, avoids the surface waste storage requirements, and contributes to the development of less energy-intensive carbon dioxide utilization and solid waste valorization methods.
- The carbonation method adopted enables the valorization of bituminous coal-derived fluidized bed fly ash with an increased calcium oxide content.
- Additional research into the various applications of carbonated fluidized bed fly ash as a cementitious material are needed to further investigate the impact of carbonation conditions and cement substitution on the properties of the selected final materials and/or by-products.

## Figures and Tables

**Table 1:** Physical and chemical properties of materials tested.

Parameter, Unit	Sample						
	1	2	3	4	5	6	7
Moisture, % *w*/*w*	<0.20	0.23	<0.20	<0.20	0.26	0.34	1.42
Carbon, % *w*/*w*	4.83	4.41	2.17	3.25	0.31	0.61	1.65
Hydrogen, % *w*/*w*	<0.11	<0.11	<0.11	<0.11	<0.11	<0.11	<0.11
Nitrogen, % *w*/*w*	<0.15	<0.15	<0.15	<0.15	<0.15	<0.15	<0.15
Sulphur, % *w*/*w*	2.88	3.10	2.85	3.35	2.53	1.02	3.13
Chlorine, % *w*/*w*	0.48	0.68	0.18	0.19	<0.07	<0.07	0.17
Chlorides, % *w*/*w*	0.43	0.63	0.17	0.18	<0.08	<0.08	0.14
Sulfates, % *w*/*w*	6.41	6.82	6.66	8.20	5.91	2.56	7.13
Free CaO, % *w*/*w*	1.41	2.63	15.30	3.02	3.36	1.16	1.06
SiO_2_, % *w*/*w*	39.31	35.20	33.44	38.21	50.73	20.11	26.22
Al_2_O_3_, % *w*/*w*	20.96	19.33	16.23	20.78	20.81	5.37	11.85
Fe_2_O, % *w*/*w*	7.86	6.89	4.10	5.91	3.82	2.84	4.40
CaO, % *w*/*w*	11.30	16.33	30.14	15.65	11.09	64.32	39.88
MgO, % *w*/*w*	2.18	1.52	1.73	2.41	1.37	1.34	1.59
Na_2_O, % *w*/*w*	2.42	1.98	0.58	0.92	0.42	0.01	1.50
K_2_O, % *w*/*w*	2.01	1.92	1.59	2.21	2.46	0.27	1.18
SO_3_, % *w*/*w*	6.34	6.99	6.71	8.38	6.25	3.00	7.82
TiO_2_, % *w*/*w*	0.83	0.70	0.69	0.86	0.90	0.25	0.47
P_2_O_5,_ % *w*/*w*	0.10	0.14	0.14	0.36	0.06	0.16	0.17
pH, % *w*/*w*	>12.0	>12.0	>12.0	>12.0	>12.0	>12.0	>12.0
Density, g/cm^3^	2.41	2.49	2.80	2.59	2.71	3.18	2.81

**Table 2:** Composition of solid state components (composites) of the injection mixtures tested.

Composite No	Composition of Solid State Components of Injection Mixtures
C1	95% *w*/*w* of carbonated fluidized bed fly ash 1 + 5% *w*/*w* of cement
C2	95% *w*/*w* carbonated fluidized bed fly ash 2 + 5% *w*/*w* of cement
C3	95% *w*/*w* of carbonated fluidized bed fly ash 3 + 5% *w*/*w* of cement
C4	95% *w*/*w* of carbonated fluidized bed fly ash 4 + 5% *w*/*w* of cement
C5 C6	92.5% *w*/*w* of carbonated fluidized bed fly ash 4 + 7.5% *w*/*w* of cement
C7 C8	97.5% *w*/*w* of carbonated fluidized bed fly ash 4 + 2.5% *w*/*w* of cement
C9 C10	92.5% *w*/*w* of carbonated fluidized bed fly ash 4 + 7.5% *w*/*w* of mineral mining binder
C11 C12	95% *w*/*w* of carbonated fluidized bed fly ash 4 +5% *w*/*w* of mineral mining binder
C13 C14	97.5% *w*/*w* of carbonated fluidized bed fly ash 4 + 2.5% *w*/*w* of mineral mining binder
O15 C16	82.5% *w*/*w* of carbonated fluidized bed fly ash 4 + 10% *w*/*w* of bottom ash + 7.5% *w*/*w* of mineral mining binder
C17 C18	85% *w*/*w* of carbonated fluidized bed fly ash 4 + 10% *w*/*w* of bottom ash + 5% *w*/*w* of mineral mining binder
C19 C20	87.5% *w*/*w* of carbonated fluidized bed fly ash 4 + 10% *w*/*w* of bottom ash + 2.5% *w*/*w* of mineral mining binder

**Table 3:** Composition of water extracts of materials tested.

Parameter, Unit	Sample						
	1	2	3	4	5	6	7
Permanganate index, mg O_2_/dm^3^	5.1	5.8	3.1	11	21	10	19
Arsenic, mg/dm^3^	<0.01	<0.01	<0.01	<0.01	<0.02	<0.01	<0.005
Barium, mg/dm^3^	0.52	0.42	0.39	0.70	0.29	0.58	0.71
Chromium, mg/dm^3^	0.023	0.030	0.055	0.140	0.008	0.390	0.075
Tin, mg/dm^3^	<0.005	<0.005	<0.005	<0.005	<0.005	<0.005	<0.005
Zinc, mg/dm^3^	<0.03	<0.03	<0.03	<0.03	<0.05	<0.03	<0.03
Cadmium, mg/dm^3^	<0.001	<0.001	<0.001	<0.001	<0.001	<0.001	<0.001
Cobalt, mg/dm^3^	<0.005	<0.005	<0.005	<0.005	<0.005	<0.005	<0.005
Copper, mg/dm^3^	<0.005	<0.005	<0.005	<0.005	<0.005	<0.005	<0.005
Molybdenum, mg/dm^3^	0.150	0.130	0.049	0.170	0.046	0.061	0.041
Nickel, mg/dm^3^	<0.005	<0.005	<0.005	<0.005	<0.005	<0.005	<0.005
Lead, mg/dm^3^	<0.005	<0.005	<0.01	<0.01	<0.02	<0.005	<0.005
Potassium, mg/dm^3^	6.33	8.80	14.50	9.50	3.54	377	126
Mercury, mg/dm^3^	<0.001	<0.001	<0.001	<0.001	<0.001	<0.001	<0.001
Sodium, mg/dm^3^	59.7	44.9	30.8	19.7	1.00	36.3	516
Chlorides, mg/dm^3^	457	635	166	129	<5	17	89
Cyanide total, mg/dm^3^	<0.005	<0.005	<0.005	<0.005	<0.005	<0.005	<0.005
Cyanide free, mg/dm^3^	<0.005	<0.005	<0.005	<0.005	<0,.05	<0.005	<0.005
Sulfates, mg/dm^3^	1650	1610	1660	1670	1690	985	56
Sulfides, mg/dm^3^	<0.05	<0.05	<0.05	<0.05	<0.18	<0.05	<0.05

**Table 4:** Radioactivity of materials tested.

Radioactive Nuclide, Bq/kg	Sample						
	1C	2C	3C	4C	5	6	7
^238^U	137	140	87.5	116	68	27.4	57
^235^U	5.8	6.1	3.6	5.1	4.3	1.6	2.6
^226^Ra	141	138	78.7	120	83.4	33.1	63.3
^228^Ra	104	90.6	59.4	88.5	61.3	17.7	42.9
^228^Th (^224^Ra)	97.6	87.4	58.5	85.7	69.7	16.3	42.1
^210^Pb	135	139	81.6	97.2	133	8.7	42.4
^40^K	562	530	384	595	532	211	341

**Table 5:** Properties of filling mixtures tested.

Filling Mixture	Composite	Water/Solids Mass Ratio,	Density, g/cm^3^	Flowability, mm	Overlaying Water, %	Setting Time, d	Slakeability	Viscosity, -	Compressive Strength, MPa		
									28 d	56 d	84 d
M1	C1	1.05	1.40	180	0.0	3	+	14.3	1.32 ± 0.09	-	-
M2	C2	1.00	1.38	195	6.9	3	+	14.4	1.31 ± 0.10	-	-
M3	C3	0.90	1.37	185	8.3	3	+	4.4	0.57 ± 0.05	-	-
M4	C4	0.90	1.44	190	5.6	2	+	6.6	1.78 ± 0.16	-	-
M5	C5	0.70	1.55	205	6.3	2	+	9.3	3.69 ± 0.18	4.78 ± 0.25	4.66 ± 0.00
M6	C6	0.90	1.48	180	6.4	2	+	6.3	2.79 ± 0.33	3.01 ± 0.32	3.37 ± 0.43
M7	C7	0.70	1.57	175	4.8	6	+	10.9	1.08 ± 0.05	1.73 ± 0.00	1.00 ± 0.09
M8	C8	0.90	1.48	175	4.0	4	+	7.3	1.30 ± 0.07	1.45 ± 0.23	2.00 ± 0.10
M9	C9	0.80	1.60	185	3.2	2	+	12.7	0.82 ± 0.02	1.57 ± 0.07	1.46 ± 0.09
M10	C10	0.90	1.48	180	2.4	3	+	8.7	0.56 ± 0.14	1.18 ± 0.04	1.31 ± 0.01
M11	C11	0.80	1.56	180	4.0	2	+	12.3	0.88 ± 0.08	1.24 ± 0.07	0.96 ± 0.02
M12	C12	0.90	1.44	185	2.4	3	+	8.5	0.40 ± 0.05	1.06 ± 0.01	1.23 ± 0.03
M13	C13	0.80	1.60	185	3.3	3	+	12.0	0.59 ± 0.08	1.36 ± 0.01	0.82 ± 0.05
M14	C14	0.90	1.48	185	3.2	3	+	8.3	0.21 ± 0.03	0.80 ± 0.08	0.80 ± 0.02
M15	C15	0.70	1.55	180	5.2	3	+	18.2	1.90 ± 0.05	2.25 ± 0.31	1.86 ± 0.10
M16	C16	0.80	1.52	175	2.8	2	+	13.8	0.96 ± 0.09	1.76 ± 0.01	1.69 ± 0.23
M17	C17	0.80	1.54	195	4.9	4	+	18.1	1.92 ± 0.01	2.21 ± 0.01	1.73 ± 0.01
M18	C18	0.90	1.44	205	8.8	3	+	13.3	0.95 ± 0.04	1.45 ± 0.14	1.77 ± 0.12
M19	C19	0.70	1.60	185	4.8	2	+	17.3	1.61 ± 0.10	2.66 ± 0.22	2.08 ± 0.18
M20	C20	0.80	1.44	210	8.8	3	+	12.8	0.84 ± 0.10	1.21 ± 0.07	1.26 ± 0.26

**Table 6:** Selected recommended parameters of injection mixtures for filling voids and goafs as well as porous and loose layers on the basis of [52].

Parameter, Unit	Injection Mixtures for Filling Voids and Goafs	Injection Mixtures for Filling Porous and Loose Layers
Flowability, mm	160–210	160–210
Setting time, d	<7	- *
Compressive strength, MPa	0.5 after 28 d	0.5 after 28 d
Slakeability_,_ %	<20	<20
Overlaying water, %	<7	<7

* The potential limitation may result from the specific technological conditions at the particular location.

**Table 7:** Selected recommended criteria for application of fine-grained mixtures in underground mining techniques on the basis of [52].

Parameter, Unit	Injection Mixtures for Grouting of Old Goafs after Roof Falling Down	Injection Mixtures for Preparation of Plugs and Packwalls	Injection Mixtures for Isolating Fire Areas	Injection Mixtures for Pit Shaft and Fore-Shaft Liquidation
Flowability, mm	210–250	160–180	160–210	160–180
Setting time, d	- *	<2	- *	- *
Compressive resistance, MPa	0.1 after 28 d	0.5 after 28 d	0.1 after 28 d	0.1 after 28 d
Slakeability, %	<80	<20	<20	<80
Overlying water_,_ %	<14	<7	<14	<7

* The potential limitation may result from the specific technological conditions at the particular location.

## Data Availability

All data generated or analyzed during the study are included in the published article.
